# PSYCHOLOGICAL IMPACT OF INCREASING HATE CRIMES AGAINST ASIAN AMERICANS AND PERCEPTIONS OF ACTIVISM DURING COVID-19

**DOI:** 10.1093/geroni/igae098.2337

**Published:** 2024-12-31

**Authors:** Eunhye Kim, Y Joon Choi, Candace Griffith

**Affiliations:** Augusta University, Augusta, Georgia, United States; Georgia State University, Atlanta, Georgia, United States; Augusta University, Augusta, Georgia, United States

## Abstract

**Objectives:**

The purpose of this study is to examine how Asian hate crimes impact Korean American older adults’ daily life and their perception of social advocacy during COVID-19.

**Background:**

In addition to the health crisis, Asian Americans have experienced more risk and challenges during the pandemic due to the explosion of anti-Asian hate crime incidents. Violence against older Asian Americans increases fear and anxiety not only for individuals directly affected but for entire Asian communities. In this regard, understanding older Asian Americans’ perception of hate crimes against Asians and their attitudes about social advocacy can help guide support and services for this population.

**Methods:**

A qualitative approach was used for this study, including semi-structured interview guides with 23 Korean American older adults. Data organization was conducted with Nvivo12, employing thematic analysis to identify key themes and sub-themes.

**Results:**

The findings showed that participants believed that rising anti-Asian hate crimes have restricted Asian American’s daily activities by increasing fear and feelings of being perpetual foreigners in American society. Participants believed that social action is necessary to change society, but they still hold passive attitudes on social action and have a lack of resources. They believed leaders and other powerful figures in the community rather than individuals should address social problems.

**Conclusion:**

Findings from this study highlight the psychological impact of anti-Asian hate crimes and the importance of expanding support for ethnic minority older adults to voice out and promoting increased advocacy for this population.

